# Attractive study of the antimicrobial, antiviral, and cytotoxic activity of novel synthesized silver chromite nanocomposites

**DOI:** 10.1186/s13065-022-00832-y

**Published:** 2022-05-27

**Authors:** Mohsen A. Sayed, Tahany M. A. Abd El-Rahman, H. K. Abdelsalam, Ahmed M. Ali, Mayar M. Hamdy, Yara A. Badr, Nada H. Abd El- Rahman, Sabah M. Abd El-Latif, Sara H. Mostafa, Sondos S. Mohamed, Ziad M. Ali, Asmaa A. H. El-Bassuony

**Affiliations:** 1 Botany and Microbiology Department, Faculty of Science, Cairo University, Giza, Egypt; 2 Basic Science Department, Higher Institute of Applied Arts 5th Settlement, New Cairo, Egypt; 3 Biotechnology Department, Faculty of Science, Cairo University, Giza, Egypt; 4 Physics Department, Faculty of Science, Cairo University, Giza, Egypt

**Keywords:** Silver chromite, Nanoparticles, Antimicrobial, Antiviral, MIC, Cytotoxic activity

## Abstract

Antibiotic resistance is a global problem. This is the reason why scientists search for alternative treatments. In this regard, seven novel silver chromite nanocomposites were synthesized and assayed to evaluate their antimicrobial, antiviral, and cytotoxic activity. Five bacterial species were used in this study: three Gram-positive (*Bacillus subtilis*, *Micrococcus luteus, *and *Staphylococcus aureus*) and two Gram-negative (*Escherichia coli* and *Salmonella enterica*). Three fungal species were also tested: *Candida albicans, Aspergillus niger,* and *A. flavus.* The MIC of the tested compounds was determined using the bifold serial dilution method. The tested compounds showed good antibacterial activity. Maximum antibacterial activity was attained in the case of 15 N [Cobalt Ferrite (0.3 CoFe_2_O_4_) + Silver chromite (0.7 Ag_0.5_Cr_2.5_O_4_)] against *M. luteus.* Concerning antifungal activity, *C.* *albicans* was the most susceptible fungal species. The maximum inhibition was recorded also in case of 15 N [Cobalt Ferrite (0.3 CoFe_2_O_4_) + Silver chromite (0.7 Ag_0.5_Cr_2.5_O_4_)]. The most promising antimicrobial compound 15 N [Cobalt Ferrite (0.3 CoFe_2_O_4_) + Silver chromite (0.7 Ag_0.5_Cr_2.5_O_4_)] was assayed for its antiviral and cytotoxic activity. The tested compound showed weak antiviral activity. The cytotoxic activity against Mammalian cells from African Green Monkey Kidney (Vero) cells was detected. The inhibitory effect against Hepatocellular carcinoma cells was detected using a MTT assay. The antimicrobial effect of the tested compounds depends on the tested microbial species. The tested compounds could be attractive and alternative antibacterial compounds that open a new path in chemotherapy.

## Introduction

Antibiotics and antimicrobial compounds which can inhibit the growth of microbes or kill them are commonly applied in microbial diseases treatment in humans. The world production of different antimicrobial compounds is 100 –200 thousand tons [1, 2]. The development of antimicrobial resistance to antibiotics led to a huge social and economic impact that causes a significant threat to our future [3]. Current treatments either are less effective or result in further acquired resistance [4]. Dangerous, antibiotic-resistant bacterial frequency increased over the past decades [5]. The emergence of bacterial resistance worldwide affects antibiotic efficacy [6]. Excessive uptake of antimicrobial compounds leads to the development of resistance to antibiotics which leads to the post-antibiotics era, where microorganisms developed multi-drug resistance [7].

Nanoparticle synthesis is a revolution that happened in all fields that attracted researchers from all branches of science [8–11]. Nanoparticles existed on Earth and man life. It can be manufactured biologically, anthropogenically, and geologically in erosion, volcanoes, fires of forests, burning of charcoal, and industry [12]. Moreover, nanoparticles can be prepared by different methods such as the solid-state method and wet methods [13–17]. The easy, low cost, rapid, save time and give high yield method is the Flash auto-combustion technique in which all the investigated samples have been prepared with it [18–22]. Magnetic nanomaterials are important in many technological applications especially biomedical applications [23–25]. As reported, there is a relation between the saturation magnetization and the antimicrobial properties in which high saturation magnetization gives strong antibacterial activity [26]. The physical and magnetic properties of silver chromites nanoparticles were studied in detail in previous work [27]. Considering the mechanism of nanoparticles, nanoparticles of silver are strong candidates as antiviral compounds. Nanoparticles attack a wide range of microbes which is futile [28]. Developments in Nanomedicine lead to evaluation and understanding of the ability of silver nanoparticles (SNPs) to be good antibiotic alternatives [29]. Nanoparticles are used to target bacteria as an alternative to antibiotics [30]. Different types of interactions of nanoparticles with bacterial cells include reactive oxygen production, the release of cation, damage of biomolecules, depletion of ATP, and interaction with membrane [31].

Many methods are used to screen the in vitro antimicrobial activity, like disk-diffusion and broth methods [32].

Generally, the cytotoxicity of the nanoparticles increased with an increase in concentration [33]. Nanoparticles can cross biological barriers and access a wide range of tissues in the body, such as the brain [34]. The risk and hazard of nanoparticles are key to be used by humans. In vivo studies is the source of information regarding its effects on the physiology of organisms [35, 36].

Silver chromite is applied in the preparation of important compounds that can be applied in many fields such as cancer diagnosis, biomedical applications, and antimicrobial activities [29, 37]. The use of nanomaterials is a suitable way to overcome microbial resistance [3].

In this work novel synthesized silver chromite nanocomposites were evaluated for their antimicrobial, antiviral, and cytotoxic activity.

## Experimental techniques

### Synthesis of nanoparticles

Seven nanocomposites were prepared by flash method with initial ingredients metal nitrates such as silver nitrate, chromium nitrate, copper nitrate, lanthanum nitrate, cobalt nitrate, iron III nitrate, and urea mixed with a small amount of distilled water with a stoichiometric ratio. Then the mixture was heated at 250 °C. The ashes were produced then the powders were ground. Samples 1 N and 2 N were a Nanocomposite of two different concentrations of lanthanum perovskite and silver chromite, where 1 N [Lanthanum perovskite (0.3LaFeO_3_) + Silver chromite (0.7Ag_0.5_Cr_2.5_O_4_)] and 2 N [Lanthanum perovskite (0.5 LaFeO_3_) + Silver chromite (0.5Ag_0.5_Cr_2.5_O_4_)]. Samples 8 N and 10 N were a Nanocomposite of two different concentrations of copper ferrite and silver chromite, where 8 N [Copper Ferrite (0.3 CuFe_2_O_4_) + Silver chromite (0.7Ag_0.5_Cr_2.5_O_4_)], and 10 N [Copper Ferrite (0.5 CuFe_2_O_4_) + Silver chromite (0.5 Ag_0.5_Cr_2.5_O_4_)]. Sample 15 N was a nanocomposite nanoparticle of the mixture of concentration 0.3 cobalt ferrite and 0.7 silver chromite, and 17 N was a Nanocomposite of two different concentrations of cobalt ferrite and silver chromite, where 15 N [Cobalt Ferrite (0.3CoFe_2_O_4_) + Silver chromite (0.7 Ag_0.5_Cr_2.5_O_4_)], and 17 N [Cobalt Ferrite (0.5CoFe_2_O_4_) + Silver chromite (0.5Ag_0.5_Cr_2.5_O_4_)]. Finally, Sample 15 was pure silver chromite (Ag_0.5_Cr_2.5_O_4_). High-resolution transmission electron microscopy was studied for the pure sample using the HRTEM model Tecnai G20, Netherlands. Moreover, the field emission scanning electron microscopy (FESEM) was studied for pure sample using SEM model quanta 250 FEG apparatus. Also, Atomic force microscopy (AFM) was studied for 8 N nanoparticles (0.3 CuFe_2_O_4_ + 0.7Ag_0.5_Cr_2.5_O_4_) using non-contact wet SPM 9600 Shimadzu.

### Biological activity

#### Materials

Fungi and bacteria were supplied by the Faculty of Science, Cairo University.

#### Isolation and identification of microorganisms

Fungal species were cultivated on a PDA medium; plates were incubated at 25 °C for 7 days. The identification was assured, according to Moubasher [38]. Bacteria were grown on nutrient agar plates. The identification was assured according to growing Bergey’s Manual of Determinative Bacteriology [39].

#### Assay of antimicrobial activity

Sabouraud medium was inoculated with 10^6^ colony forming units/ml (CFU/ ml) of microorganisms. Then, discs (6 mm in diameter) containing 100 µg/ml of the test compound were added to the agar surface. The Petri plates were incubated at 27 °C for 5 days. The diameters of inhibition growth zones were measured. Three fungal species: *Aspergillus flavus, A. niger,* and *Candia albicans,* were used in this study. Tioconazole (100 µg/ml) was used as a control [40]. Five bacterial species were assayed; three Gram-positive species (*Staphylococcus aureus,* *Micrococcus luteus,* and *Bacillus subtilis*) and two Gram-negative species (*Salmonella enterica* and *Escherichia coli*) were added at concentration 10^6^ (CFU/ml) to Mueller–Hinton Agar before solidification. Plates were incubated at 35 °C for 2 days. Ampicillin (100 µg/ml) was used as a control [41].

#### Relative activity

The ratio between the activity of a sample of interest and the activity of the control sample.

#### Minimum Inhibitory Concentration

The MIC of the assayed compounds was determined [42]. The serial dilution method evaluated the inhibitory effect of the different compounds on bacterial growth. The studied concentrations were 2000, 1000, 500, 250, 125, 62.5, 31.25, 15.62, 7.8, 3.9, 1.9, and 0.9 µg/ml. The compounds were dissolved in dimethyl sulfoxide. Mueller–Hinton broth and Sabouraud liquid medium were used as culture media for bacteria and fungi, respectively. Test inoculums of 10^4^ /CFU were applied.

### Antiviral assay

#### Viral propagation

The cytopathogenic HAV (HM175 strain) (ATCC VR-1402) of Hepatitis A Virus was assayed in confluent Vero cells [43], and it was counted by determination of the 50% infectious dose (TCID50) using the Spearman-Karber method [44].

#### Antiviral activity

It was done using the Regional Center for Mycology and Biotechnology (RCMB, Al-Azhar University, Cairo, Egypt). This assay was done by the MTT method [45]. Amantadine was used as a control. The viability of cells was determined [46]. The viral inhibition rate was: ([(A − B)/(C − B)] × 100%) where A, B, and C are the absorbance of the tested compounds, the absorbance of the virus control, and the absorbance of the cell control, respectively.

### Cytotoxicity

#### Mammalian cell line

Vero cells (derived from the kidney of African green monkey) (ATCC, Manassas, VA, USA). Vero cells were propagated in Dulbecco’s modified Eagle medium [47]. Viable cell yield was determined by a MTT [46]. The optical density was measured at 590 nm with the microplate reader (Sunrise, TECAN, Inc, USA). The viability percentage was calculated as [(ODt/ODc)] × 100%, where ODt is the optical density of the tested sample and ODc is the optical density of untreated cells.

#### Hepatocarcinoma cells

HepG-2cells (ATCC, Rockville, MD), and the chemicals Used: Dimethyl sulfoxide (DMSO), MTT, and trypan blue dye (Sigma, St. Louis, Mo., USA). Fetal Bovine serum, DMEM, RPMI-1640, HEPES buffer solution, L-glutamine, gentamycin, and 0.25% Trypsin–EDTA (Lonza, Belgium).

##### Cell line propagation

The cells were grown on RPMI-1640 medium supplemented with 10% inactivated fetal calf serum and 50 µg/ml gentamycin. The cells were maintained at 37 ºC. The optical density was measured at 590 nm with the microplate reader (Sunrise, TECAN, Inc, USA). The viability percentage was calculated as [(ODt/ODc)] × 100%. IC50 is the concentration needed to cause toxic effects in 50% of intact cells (San Diego, CA. USA) [48].

##### Statistical analysis

Data were analyzed using SPSS software version 22. According to Kolmogorov-Smirnova and Shapiro–Wilk tests, data was normally distributed within groups. Accordingly, parametric analysis was applied for the statistical analysis of data. ANOVA was utilized to study the effect of treatment on the studied parameters. Duncan’s test was utilized to study the similarity among the studied groups. An Independent t-test was applied to estimate the statistical difference between the control and 15 N groups. Data were presented as mean (n = 3) ± standard deviation.

## Results

### HRTEM analysis

**Fig. 1:**
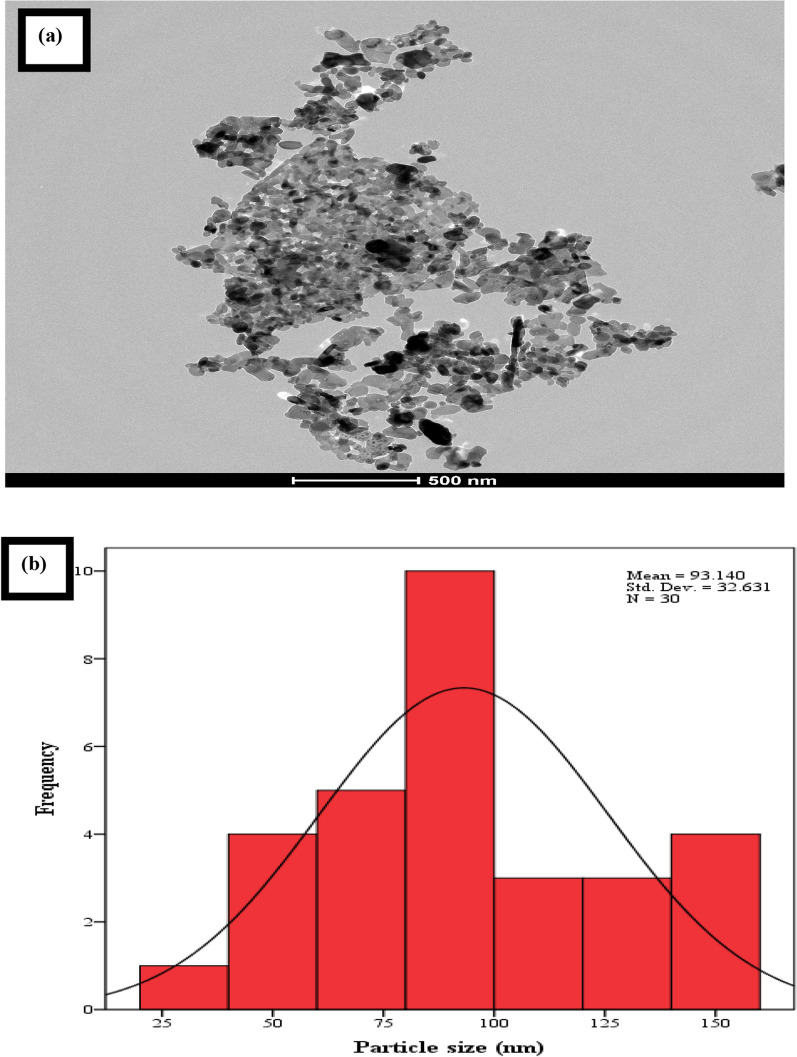
**a** HRTEM micrograph. **b** Histogram of average particle size of Ag_0.5_Cr_2.5_O_4_ nanoparticle

Figure 1a shows the morphology of the pure sample (Ag_0.5_Cr_2.5_O_4_) using High-Resolution Transmission Electron Microscopy to assure that the sample was in the nanoscale range. The nanoparticles showed an agglomeration due to no surfactant being added during the preparation method. Moreover, the smaller the particles, the easily agglomerated to each other. Figure 1b shows the histogram of the average particle size estimated from the HRTEM micrograph and shows that the nanosized of the histogram was 93.14 nm.

The polydispersity index (P) was calculated using the following formula:

1 $$P= \frac{standard\;deviation (\sigma )}{averave\;radius\;of\;the\;nanoparticles ({R}_{av})}$$

The polydispersity index showed that the pure sample gave a 0.35 value.

### FESEM analysis

**Fig. 2:**
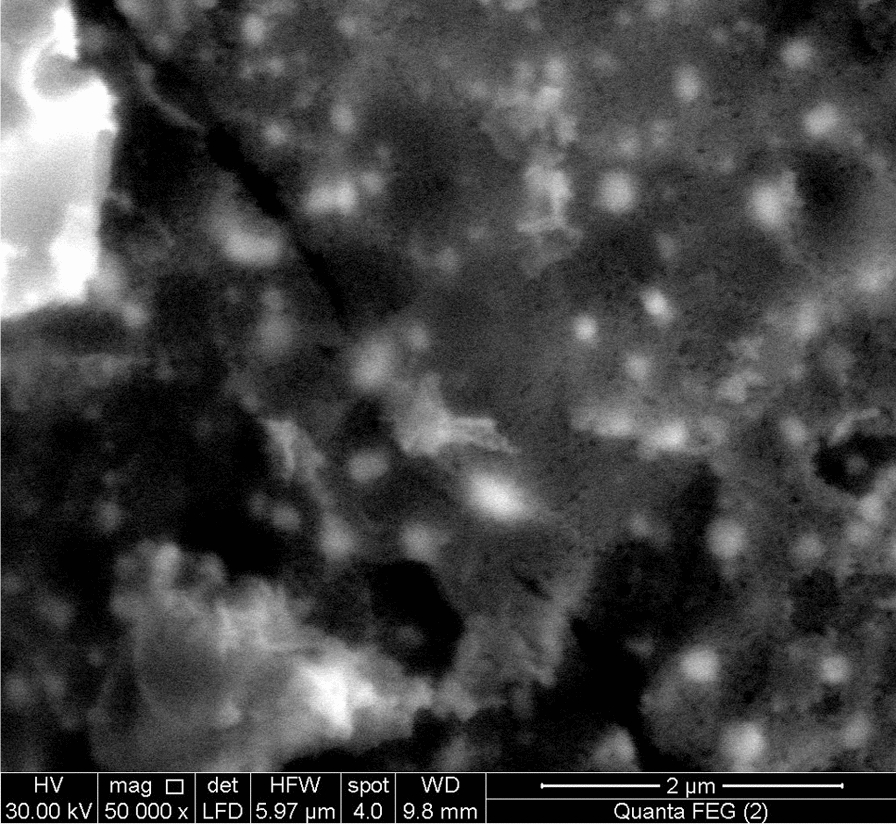
FESEM image of Ag_0.5_Cr_2.5_O_4_ nanoparticle

Figure 2 shows the field emission scanning electron microscopy (FESEM) of Ag_0.5_Cr_2.5_O_4_ nanoparticles. The figure showed that the sample in the nanosized with spherical shape and agglomeration due to the absence of surfactant during the preparation method.

### AFM analysis

**Fig. 3:**
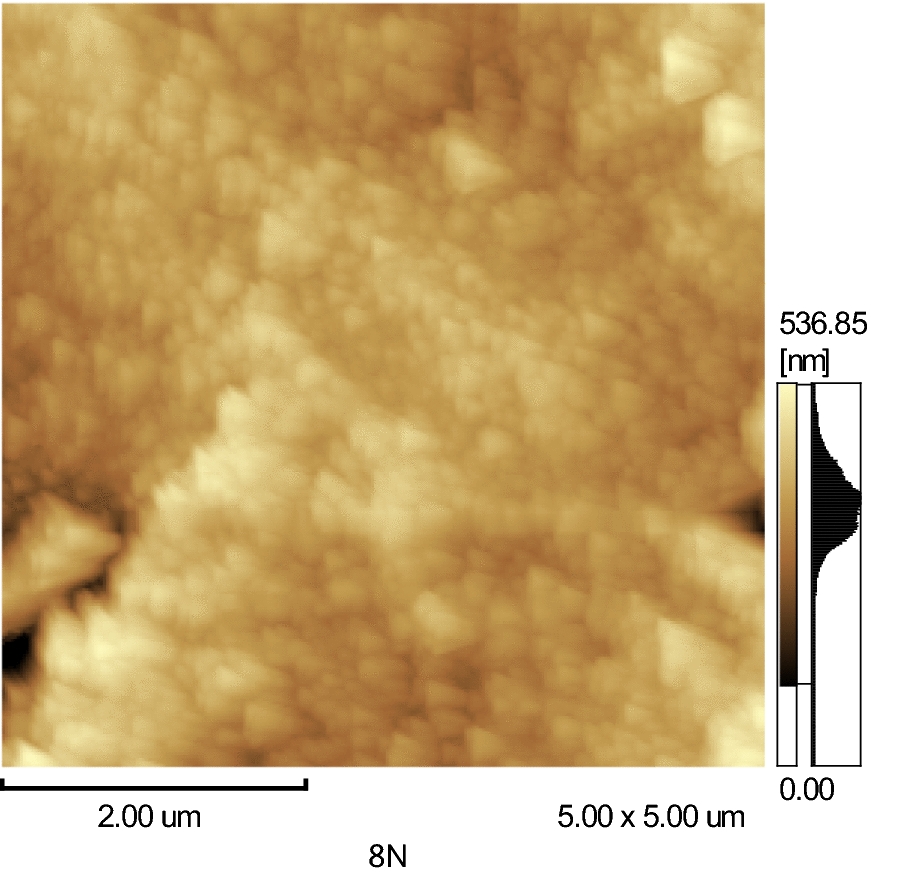
Two dimensional AFM image of 8 N nanoparticles (0.3 CuFe_2_O_4_ + 0.7 Ag_0.5_Cr_2.5_O_4_)

Figure 3 shows the atomic force microscopy (AFM) of 8 N nanoparticles (0.3 CuFe_2_O_4_ + 0.7 Ag_0.5_Cr_2.5_O_4_). The micrograph showed the size of the nanoparticles in the nano shape with agglomeration.

### Antibacterial activity

Seven nanoparticle compounds were assayed against five bacterial species: three Gram-positive (*Bacillus subtilis*, *Micrococcus luteus, *and *Staphylococcus aureus*) and two Gram-negative (*Escherichia coli* and *Salmonella enterica*).

**Fig. 4:**
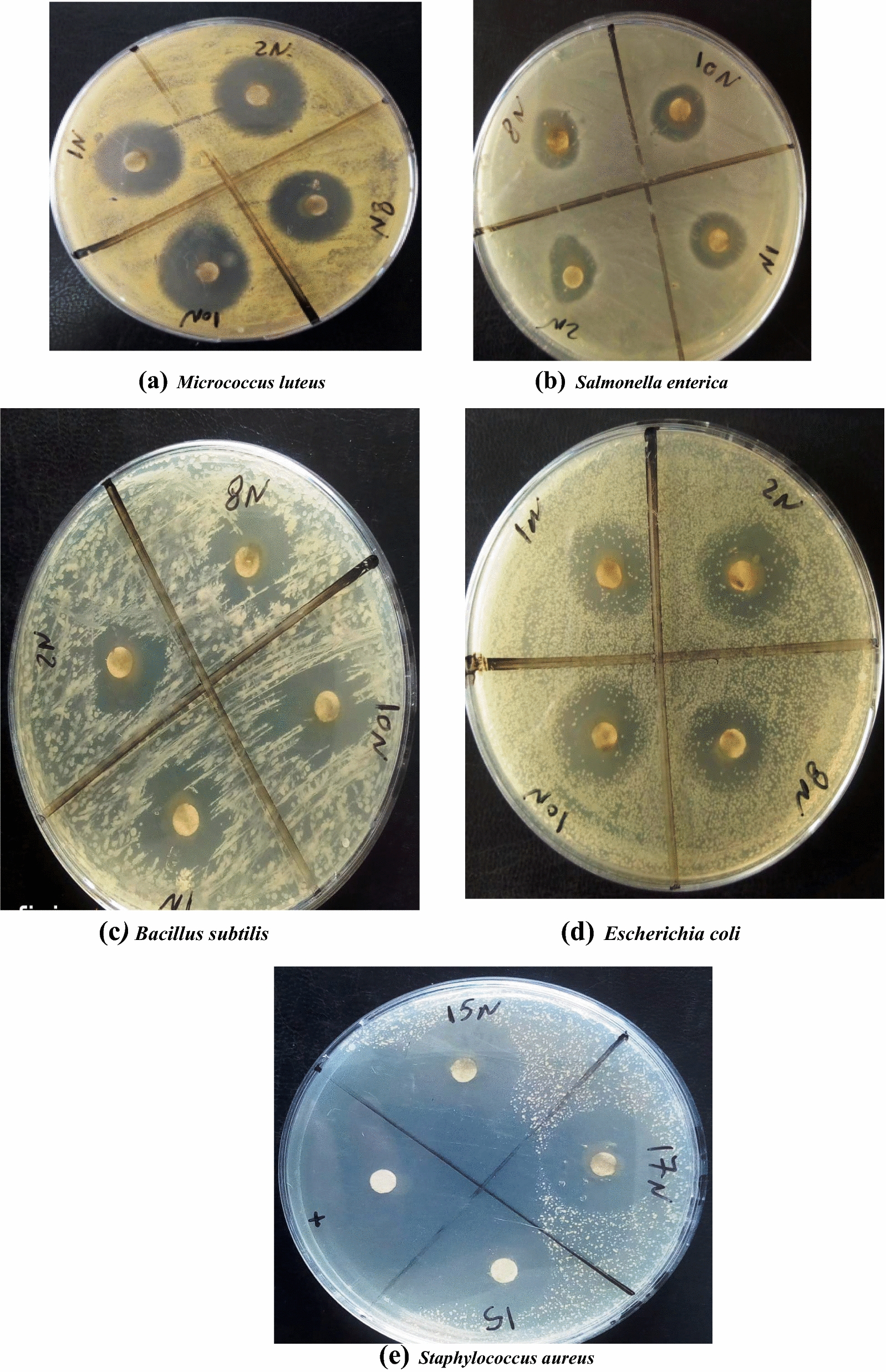
Antibacterial activity of some synthesized nanoparticle compounds against *Micrococcus Leteus, Salmonella entreica, Bacillus subtilis, Escherichia coli,* and *Staphylococcus aureus*

Maximum inhibition zone diameter (29 mm) was attained in the case of compound 15 N against *M. luteus*. Other tested species recorded lower inhibition zone diameter. The minimum inhibition zone (11 mm) was shown in the case of compound 8 N against *S. enterica* (Table 1; Fig. 4).

**Table 1:** Antibacterial activity of the synthesized nanoparticle compounds against *Bacillus subtilis, Escherichia coli, Micrococcus Leteus, Staphylococcus aureus, and Salmonella entreica*

Synthesized Nano particles	Inhibition zone diameter (mm)				
	*Bacillus* *subtilis*	*Escherichia* *coli*	*Micrococcus* *luteus*	*Staphylococcus* *aureus*	*Salmonella* *enterica*
Ampicillin (control)	46.67 ± 0.58^C^	30.83 ± 0.76.^D^	6.00 ± 1.00.^A^	64.00 ± 2.00.^D^	28.00 ± 1.99.^C^
1 N	16.00 ± 1.00^B^	15.00 ± 0.50.^C^	26.00 ± 1.00.^EF^	24.00 ± 0.98.^C^	13.67 ± 0.58.^B^
2 N	12.67 ± 0.58^A^	14.60 ± 0.72.^B^	29.00 ± 1.00.^G^	2.13 ± 0.23.^A^	13.00 ± 0.97.^AB^
8 N	15.33 ± 0.57^B^	13.07 ± 0.50.^A^	21.33 ± 0.58.^B^	22.73 ± 0.81.^BC^	11.67 ± 1.15.^A^
10 N	16.33 ± 0.58^B^	15.97 ± 0.45.^C^	25.00 ± 0.99.^DE^	2.17 ± 0.29.^A^	13.00 ± 1.00.^AB^
15 N	16.67 ± 1.53^B^	12.00 ± 0.30.^A^	27.00 ± 1.00.^F^	22.00 ± 1.00.^B^	14.03 ± 0.55.^B^
17 N	15.00 ± 1.00^B^	12.00 ± 0.96.^A^	24.33 ± 0.59.^D^	23.67 ± 0.58.^BC^	14.67 ± 0.58.^B^
15	15.00 ± 0.99^B^	13.00 ± 0.98.^A^	23.00 ± 1.00.^C^	22.00 ± 1.00.^B^	13.00 ± 0.50.^AB^
	F_7, 16_ = 420.77, P < 0.000	F_7, 16_ = 239.38, P < 0.000	F_7, 16_ = 184.05, P < 0.000	F_7, 16_ = 1077.85, P < 0.000	F_7, 16_ = 78.09, P < 0.000

Data are displayed as mean (n = 3) ± standard deviation

According to Duncan’s test, in the same column, means marked with the same superscript letters are insignificantly different (P > 0.05), whereas those marked with different ones are significantly different (P < 0.05). P < 0.000: represent significant effect

The highest relative activity (53.57%) was achieved in the case of 17 N against *S. enterica*, followed by 10 N against *E. coli* (52.38%) and (50%) in the case of 15 N against *S. enterica. Salmonella* e*nterica* was significantly inhibited by all tested nanoparticle compounds, while *Bacillus* *subtilis* was the most resistant bacterial species to the assayed compounds (Table 2).

**Table 2:** Relative activity (%) of the synthesized Nanoparticle compounds against *Bacillus subtilis, Escherichia coli, Micrococcus Leteus, Staphylococcus aureus, and Salmonella entreica*

Nanoparticle compounds	Relative activity (RA)%				
	*Bacillus* *subtilis*	*Escherichia* *coli*	*Micrococcus* *luteus*	*Staphylococcus* *aureus*	*Salmonella* *enterica*
Ampicillin	100.00 ± 0.00^E^	100.00 ± 0.00^H^	100.00 ± 0.00^G^	100.00 ± 0.00^D^	100.00 ± 0.00^F^
1 N	34.63 ± 1.00^C^	47.60 ± 0.92^F^	43.33 ± 0.35^D^	37.50 ± 0.50^C^	50.33 ± 1.53^D^
2 N	29.30 ± 0.95^A^	44.40 ± 0.40^E^	48.30 ± 1.10^F^	31.30 ± 0.66^A^	46.46 ± 0.50^B^
8 N	32.52 ± 0.30^B^	42.80 ± 0.20^D^	35.00 ± 1.00^A^	33.87 ± 0.75^B^	39.20 ± 1.01^A^
10 N	35.40 ± 0.40^C^	53.20 ± 0.25^G^	41.43 ± 1.06^C^	31.40 ± 0.53^A^	46.13 ± 1.03^B^
15 N	36.90 ± 0.36^D^	39.60 ± 0.60^B^	45.00 ± 1.00^E^	34.37 ± 0.21^B^	50.00 ± 1.00^D^
17 N	32.60 ± 0.70^B^	38.00 ± 1.00^A^	42.50 ± 0.50^D^	37.50 ± 0.50^C^	53.50 ± 0.50^E^
15	35.60 ± 0.92^C^	41.20 ± 0.31^C^	39.10 ± 0.45^B^	34.27 ± 1.05^B^	48.06 ± 0.61^C^
	F_7, 16_ = 3658.13, P < 0.000	F_7, 16_ = 3923.59, P < 0.000	F_7, 16_ = 2124.81, P < 0.000	F_7, 16_ = 4477.54, P < 0.000	F_7, 16_ = 1377.75, P < 0.000

Data are displayed as mean (n = 3) ± standard deviation

According to Duncan’s test, in the same column, means marked with the same superscript letters are insignificantly different (P > 0.05), whereas those marked with different ones are significantly different (P < 0.05). P < 0.000: represent significant effect

### Antifungal activity

**Fig. 5:**
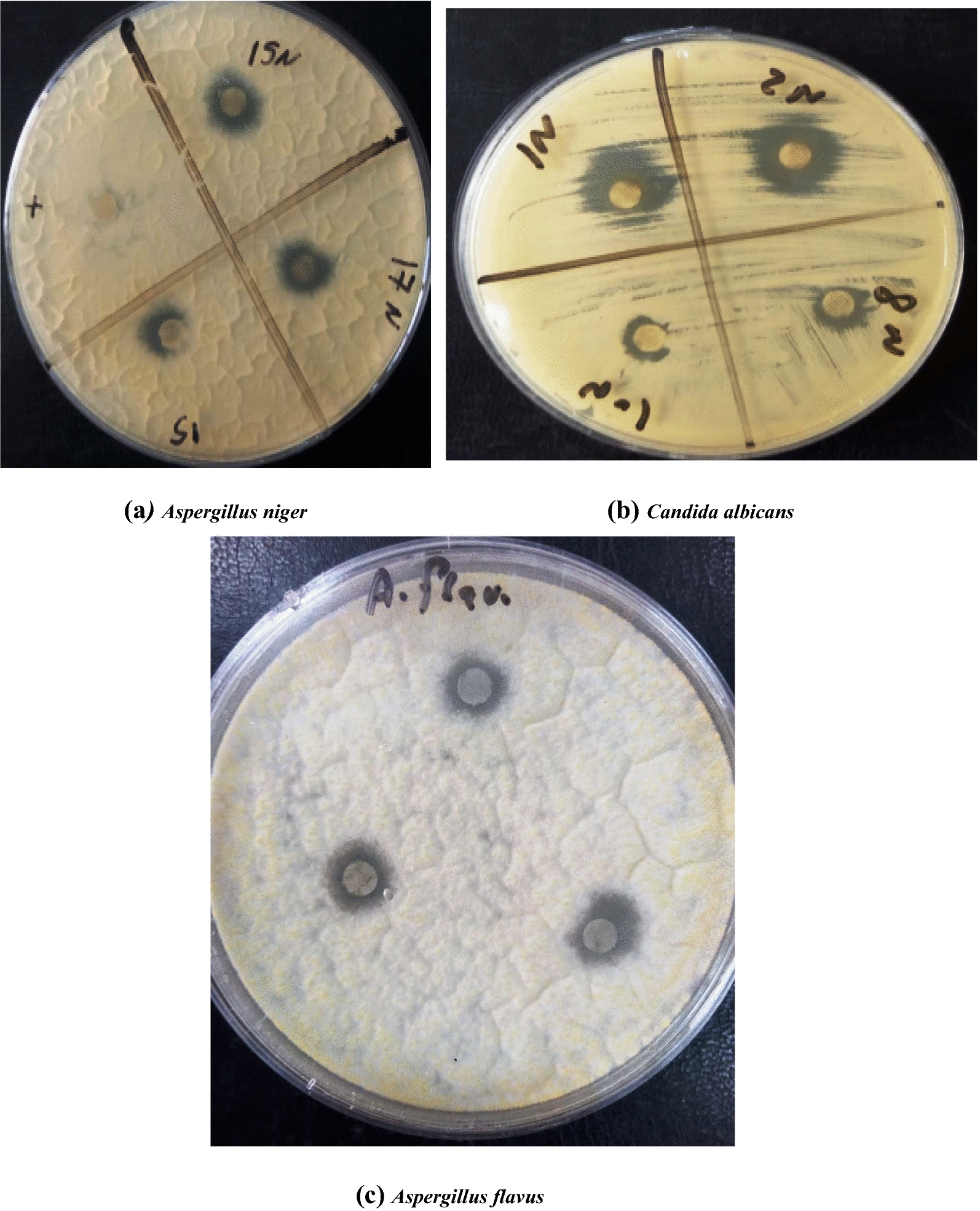
Antifungal activity of the synthesized nanoparticle compounds against *Aspergillus niger, Candida albicans,* and *Aspergillus flavus*

The seven nanoparticle compounds were assayed for their antifungal activity against three fungal species: *Candida albicans, Aspergillus flavus,* and *A. niger. C*ompounds showed significant antifungal activity against *C.* *albicans*, where 7–14 mm inhibition zone diameters were recorded. The maximum inhibition zone was attained by compound 2 N, while the minimum inhibition zone (7 mm) was recorded in the case of compound 10 N. Weak antifungal activity was recorded in the case of *Aspergillus flavus* and *A. niger *(Table 3; Fig. 5).

**Table 3:** Antifungal activity of the synthesized nanoparticle compounds against *Aspergillus flavus, Aspergillus niger, and Candida albicans*

Nanoparticle compounds	Inhibition zone diameter (mm)		
	*Aspergillus flavus*	*Aspergillus niger*	*Candida albicans*
Tioconazole (control)	0.00 ± 0.00^A^	0.00 ± 0.00^A^	25.00 ± 1.00^D^
1 N	9.00 ± 1.00^D^	11.33 ± 0.58^D^	13.00 ± 1.00^BC^
2 N	8.00 ± 0.50^C^	11.67 ± 0.58^D^	14.00 ± 1.00^C^
8 N	7.07 ± 0.50^B^	11.07 ± 0.50^D^	7.93 ± 0.12^A^
10 N	7.00 ± 0.20^B^	8.67 ± 0.58^B^	7.73 ± 0.81^A^
15 N	9.00 ± 0.30^D^	11.00 ± 0.50^D^	11.67 ± 0.76^B^
17 N	6.93 ± 0.50^B^	10.00 ± 1.00^C^	8.53 ± 0.50^A^
15	7.00 ± 0.50^B^	11.67 ± 0.58^D^	8.67 ± 0.58^A^
	F_7, 16_ = 92.28, P < 0.000	F_7, 16_ = 131.27, P < 0.000	F_7, 16_ = 164.42, P < 0.000

Data are displayed as mean (n = 3) ± standard deviation

According to Duncan’s test, in the same column, means marked with the same superscript letters are insignificantly different (P > 0.05), whereas those marked with different ones are significantly different (P < 0.05). P < 0.000: represent significant effect

Concerning the relative activity, it was found that the highest relative activity (52%, 58%, and 44%) was achieved against *C. albicans* in the case of 1 N, 2 N., and 15 N, respectively (Table 4).

**Table 4:** Relative activity (%) of the synthesized Nanoparticle compounds against *Aspergillus flavus, Aspergillus niger, and Candida albicans*

Nanoparticle compounds	Relative activity (RA) %		
	*Aspergillus flavus*	*Aspergillus niger*	*Candida albicans*
Tioconazole	8.10 ± 0.10	9.11 ± 0.20	100.00 ± 0.00^G^
1 N	4.20 ± 0.22	7.20 ± 0.10	52.00 ± 1.99^E^
2 N	5.22 ± 0.13	7.10 ± 0.11	57.67 ± 1.53^F^
8 N	5.10 ± 0.21	8.20 ± 0.58	32.00 ± 1.00^B^
10 N	3.30 ± 0.11	5.00 ± 0.00	28.33 ± 0.58^A^
15 N	2.20 ± 0.40	3.40 ± 0.52	44.17 ± 0.76^D^
17 N	3.11 ± 0.10	4.20 ± 0.21	31.67 ± 0.58^B^
15	5.00 ± 0.21	6.00 ± 0.10	36.00 ± 2.00^C^
	F_7, 16_ = 82.18, P < 0.000	F_7, 16_ = 101.17, P < 0.000	F_7, 16_ = 1057.48, P < 0.000

Data are displayed as mean (n = 3) ± standard deviation

According to Duncan’s test, in the same column, means marked with the same superscript letters are insignificantly different (P > 0.05), whereas those marked with different ones are significantly different (P < 0.05). P < 0.000: represent significant effect

### Minimum inhibitory concentration

**Fig. 6:**
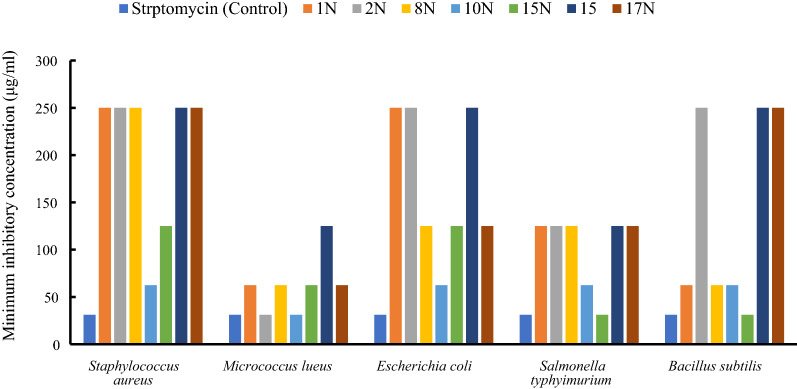
Minimum inhibitory concentrations of the nanoparticle compounds against bacterial species (*Micrococcus Leteus, Salmonella entreica, Bacillus subtilis, Escherichia coli,* and *Staphylococcus aureus)*

The data shows that the least MIC (31.25 µg/ml) was obtained in the case of 10 N and 2 N against *M. luteus*; and compound 15 N against *S. enterica* and *B. subtilis *(Table 5; Fig. 6).

**Table 5:** Minimum inhibitory concentrations of the nanoparticle compounds against *Staphylococcus aureus, Micrococcus Leteus, Escherichia coli, Salmonella typhimurium, and Bacillus subtilis*

Nanoparticle compounds	Minimum Inhibitory Concentration (MIC) (μg/ml)				
	*Staphylococcus* *aureus*	*Micrococcus* *luteus*	*Escherichia* *coli*	*Salmonella* *typhimurium*	*Bacillus* *subtilis*
Ampicillin (control)	31.25	31.25	31.25	31.25	31.25
1 N	250	62.50	250	125	62.50
2 N	250	31.25	250	125	250
8 N	250	62.50	125	125	62.5
10 N	62.50	31.25	62.50	62.50	62.5
15 N	125	62.50	125	31.25	31.25
15	250	125	250	125	250
17 N	250	62.5	125	125	250

### Antiviral assay

**Fig. 7:**
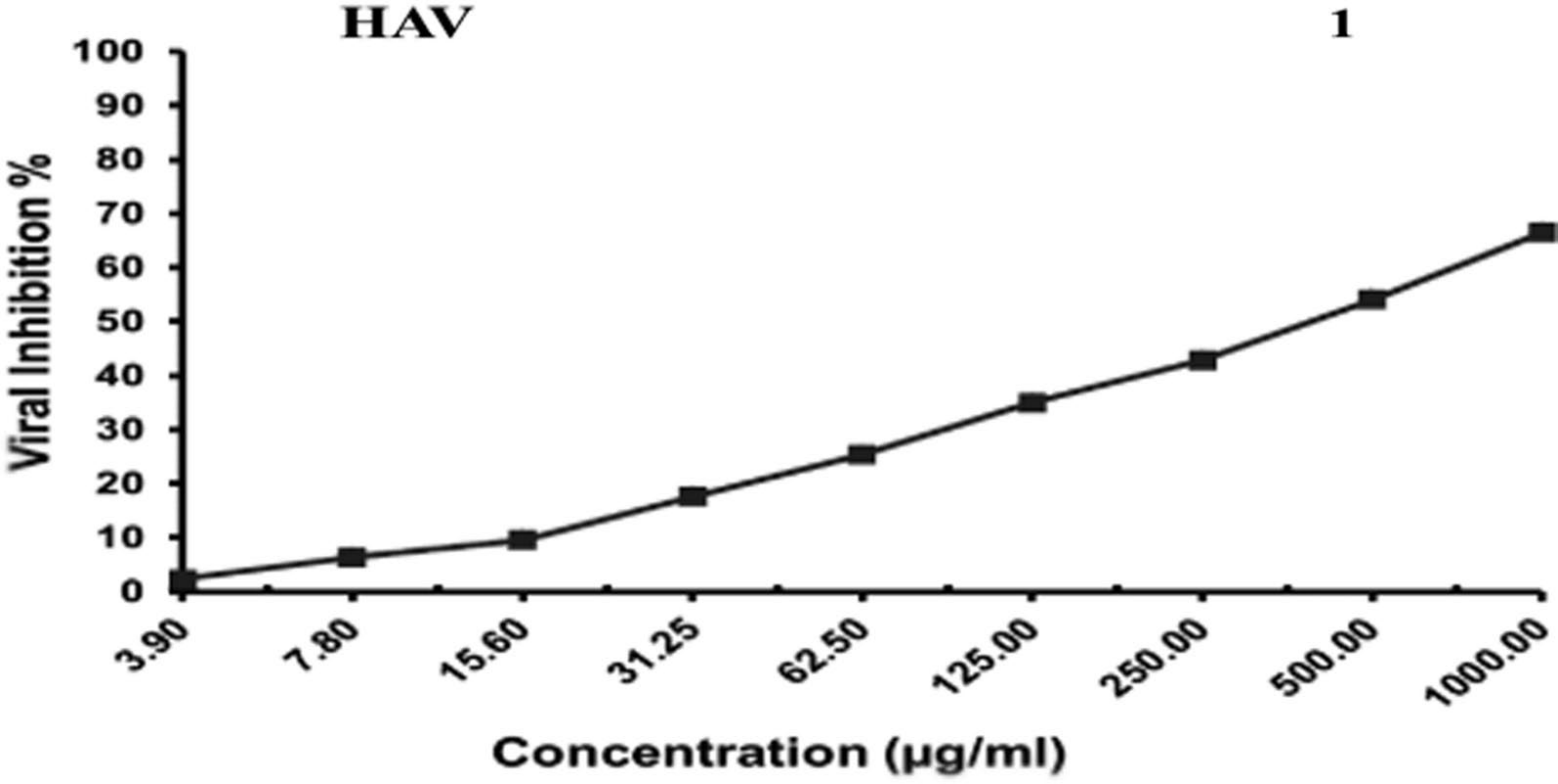
The inhibitory activity of the tested sample against Hepatitis A Virus

Based on the results of antimicrobial activity of the tested compounds, compound 15 N was chosen to evaluate its antiviral activity against the Hepatitis A virus. The tested compound showed weak antiviral activity compared to Amantadine (control). The inhibitory activity of the tested sample against the hepatitis A virus was detected under these experimental conditions with a 50% effective concentration (EC50) = 408.14 ± 21.62 µg/ml. The dose inhibited 50% (EC50) was estimated (Table 6; Fig. 7).

**Table 6:** Antiviral activity of compound 15 N against Hepatitis A virus

Treatment	MNCC (µg/ml)	Antiviral effect on HAV (%)	Antiviral effect on HAV (Qualitative)#	Antiviral Efficiency		
				EC50	CC50	SI
Amantadine (control)	130	86.91 ± 5.57	++++	8.48 ± 0.50	325.61 ± 16.9	38.39
15 N	60	7.24 ± 0.64*	+	408.14 ± 21.60*	280.36 ± 19.3*	0.69

Data is displayed as mean (n = 3) ± standard deviation

^*^: represents significant difference (P < 0.05), as compared to the corresponding controls, according to independent t-test. #Where: (+): Weak antiviral activity (1- < 25%), and (++++): Excellent antiviral activity (75–100%)

Where: ( +): Weak antiviral activity (1– < 25%), and (++++): Excellent antiviral activity (75–100%)

### Cytotoxicity test

#### Against VERO cell line

**Fig. 8:**
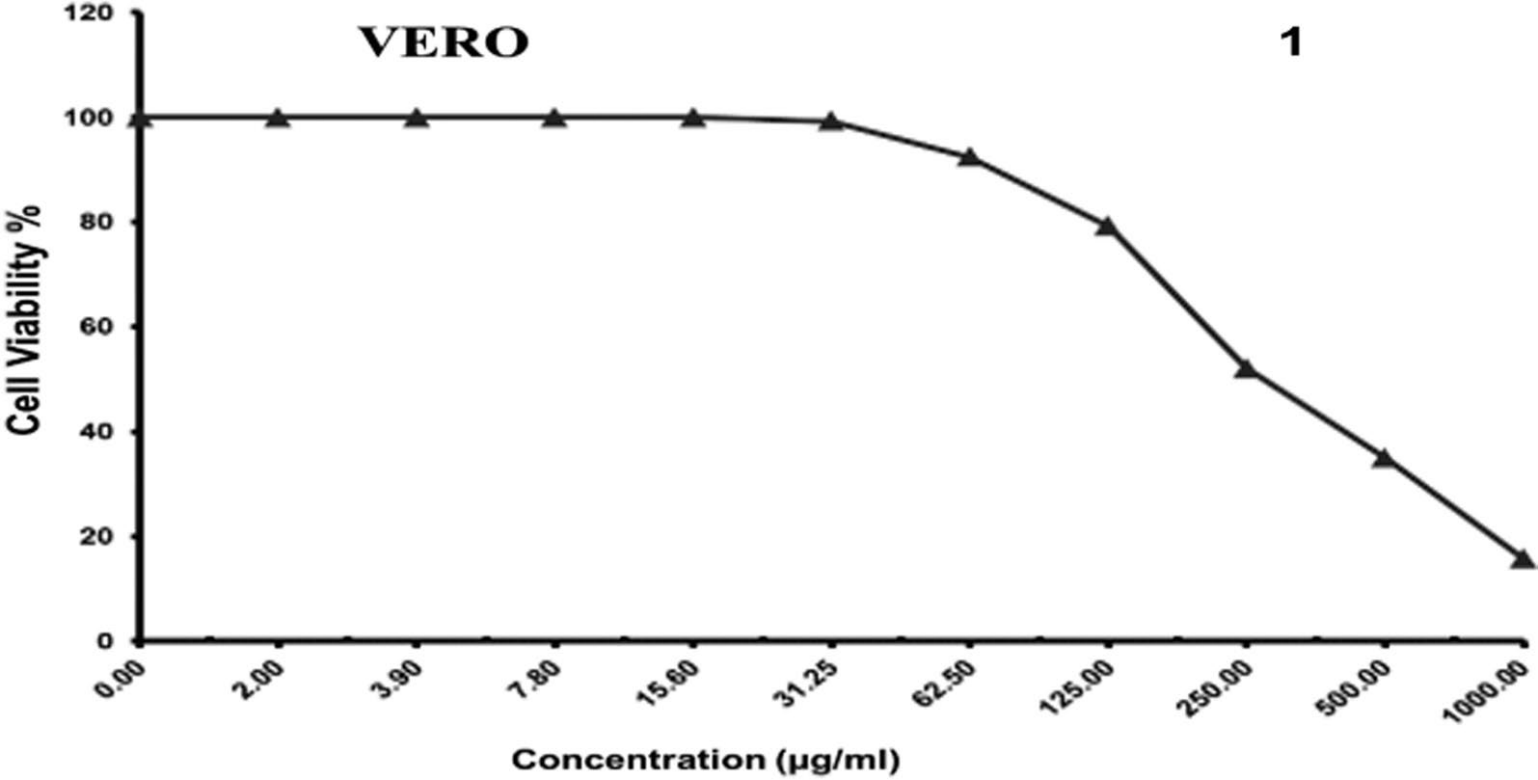
Evaluation of cytotoxicity against Mammalian cells from African Green Monkey Kidney (Vero) cell line

The Cytotoxic activity of the synthesized nanoparticles was assayed against Mammalian cells from African Green Monkey Kidney (Vero) cells was detected under these experimental conditions with 50% cell cytotoxic concentration (CC50) = 280.4 ± 19.3 µg/ml. The results showed that the higher the sample concentration the greater the cytotoxicity. Concentrations less than 31.25 µg/ml of the test compound showed no cytotoxicity (Table 7; Fig. 8).

**Table 7:** The cytotoxic activity against Mammalian cells from African Green Monkey Kidney (Vero) cells

Sample conc. (µg/ml)	Viability %	Cytotoxic %
1000	15.67 ± 2.51^A^	84.33
500	34.95 ± 3.89^B^	65.05
250	52.08 ± 2.46^C^	47.92
125	79.23 ± 2.35^D^	20.77
62.5	92.37 ± 1.27^E^	7.63
31.25	99.25 ± 0.41^F^	0.75
15.6	100.00 ± 0.00^F^	0
7.8	100.00 ± 0.00^F^	0
3.9	100.00 ± 0.00^F^	0
2	100.00 ± 0.00^F^	0
0	100.00 ± 0.00^F^	0
	F_10, 22_ = 899.76, P < 0.000	

Data are displayed as mean (n = 3) ± standard deviation

According to Duncan’s test, in the same column, means marked with the same superscript letters are insignificantly different (P > 0.05), whereas those marked with different ones are significantly different (P < 0.05). P < 0.000: represent significant effect

#### Cytotoxicity against hepatocellular carcinoma

**Fig. 9:**
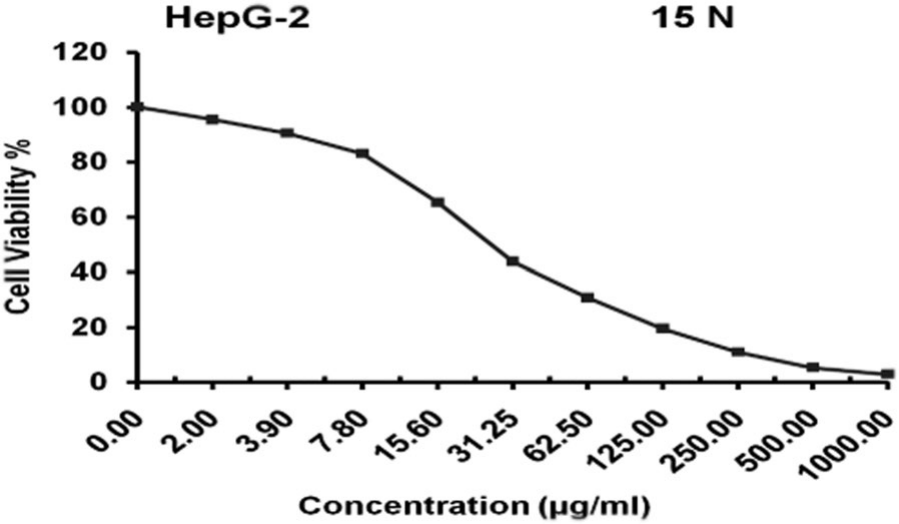
Evaluation of cytotoxicity against Hepatocellular carcinoma cells *(HepG-2)* cell line

Inhibitory activity against Hepatocellular carcinoma cells was detected using MTT assay under these experimental conditions with (IC50 = 26.7 ± 2.31 µg/ml) and this was for the compound (15 N). The results indicated that low concentrations of the tested compound (greater than 15.6 µg/ml) showed promising anticancer activity against the tested hepatocellular carcinoma cells (Table 8; Fig. 9).

**Table 8:** Inhibitory activity against Hepatocellular carcinoma cells was detected using MTT assay

Sample conc. (µg/ml)	Viability %	Inhibitory %
1000	2.85 ± 0.41^A^	97.15
500	5.31 ± 0.17^A^	94.69
250	10.86 ± 0.48^B^	89.14
125	19.47 ± 0.91^C^	80.53
62.5	30.69 ± 1.43^D^	69.31
31.25	43.76 ± 2.59^E^	56.24
15.6	65.19 ± 3.35^F^	34.81
7.8	83.04 ± 1.42^G^	16.96
3.9	90.47 ± 0.29^H^	9.53
2	95.31 ± 0.57^I^	4.69
0	100.00 ± 0.00^J^	0
	F_10, 22_ = 2044.57, P < 0.000	

Data are displayed as mean (n = 3) ± standard deviation

According to Duncan’s test, in the same column, means marked with the same superscript letters are insignificantly different (P > 0.05), whereas those marked with different ones are significantly different (P < 0.05). P < 0.000: represent significant effect

## Discussion

Antimicrobial resistance is one of the public health problems; it has a significant effect on the world. Therapeutic options to treat infectious diseases are limited due to antimicrobial resistance [49]. The misuse and overuse of antimicrobial compounds is a global phenomenon that increases the levels of antimicrobial compounds in the ecosystem and the rates of their spread [50]. The abuse of antimicrobial compounds and improper control of diseases led to the emergence of resistant microbes that are a major threat to the world's health. Therefore, research and development of new antimicrobial compounds to mitigate antibiotic resistance are imperative [51].

Nanoparticles have numerous properties, including antimicrobial activity against many microbes. The antimicrobial nanoparticle compounds can be used to overcome antibiotic resistance [52]. Antimicrobial nanoparticles such as silver nanoparticles have been utilized in biocides for over 120 years [53]. The technique used to prepare nanoparticles should be efficient, low cost, high yield, and produce very fine nanoparticles [54]. The XRD technique initially characterized the synthesized nanoparticles, which confirmed their crystalline nature. Moreover, the XRD analysis of Ag_0.5_Cr_2.5_O_4_ was discussed in detail in our previous study [27] and showed the formation of a single-phase spinel structure of the nano sample with a crystallite size of 72.6 nm. The morphology was studied using HRTEM, FESEM, and AFM to confirm that the nanoparticles of the samples were in the nanoscale range. Also, the micrograph and the histogram in Fig. 1 assured that the pure sample (Ag_0.5_Cr_2.5_O_4_) in the nanoscale range (93.14 nm) with a polydispersity index of 0.35 due to the aggregation of the nanoparticles. Moreover, the physical and magnetic properties are detailed in previous work [27].

Nanoparticles synthesized at low temperature have a small size which enables them to penetrate the cell wall and the cell membrane of the microbial cell to damage the internal organelles and molecules, causing inhibition of growth and even death [55]. The tested compounds showed significant antibacterial activity. As the concentration of nanoparticle compounds increased, the diameter of the inhibition zone increased. Gram-positive bacteria were more susceptible to the tested compounds than Gram-negative bacteria due to the absence of an outer membrane. Maximum antibacterial activity was attained in the case of 15 N [cobalt ferrite (0.3 CoFe_2_O_4_) + silver chromite (0.7 Ag_0.5_Cr_2.5_O_4_)] against *M. luteus.* The synthesized material exhibits strong bacteriostatic properties against *E. coli* at a concentration of nanoparticles of silver oxide of more than 0.01% [56]. The mode of antibacterial activity of metals includes disruption of enzyme function [57], reactive oxygen production [58], disruption of the membrane, prevention of absorption of essential elements [59], and genotoxic activity [60, 61].

The tested compounds showed significant antifungal activity. *C.* *albicans* was the most susceptible fungal species. The maximum inhibition was recorded also in case of 15 N [cobalt ferrite (0.3 CoFe_2_O_4_) + silver chromite (0.7 Ag_0.5_Cr_2.5_O_4_)]. The dispersion of silver nanoparticles in a polymer matrix enhances antibacterial efficacy by the regulated release of Ag^+^ cations, which may considerably limit infectious agent transmission [62–65].

The antiviral activity of the tested compound showed weak antiviral activity. This contrasted with Ting et al.'s conclusion that the antiviral impact of the as-prepared GO-AgNPs nanocomposites on virus replication was studied [66]. The findings suggested that exposure to GO-AgNPs nanocomposites was capable of suppressing PRRSV infection. Concerning the cytotoxicity test; it was found that the tested compounds showed low cytotoxicity against normal cells. When the concentration of silver oxide nanoparticles is less than 0.1 percent, the BS/silver oxide NPs show no harmful impact on eukaryotic cell cultures. The use of the resultant silver oxide nanoparticle composite as a reusable dry disinfectant is justified by its low toxicity and bacteriostatic activity, which are comparable to those of the medicinal alloy nitinol [55]. However, the antitumor efficacy of the silver chromite nanoparticles studied was encouraging. The biological activity of the produced Ni-Zn chromites was evaluated using Hela cell lines [67] and gave promising results. The cytotoxic activity against Mammalian cells from African Green Monkey Kidney (Vero) cells was detected. The inhibitory activity against Hepatocellular carcinoma cells was detected using a MTT assay. The most promising antimicrobial compound 15 N [cobalt ferrite (0.3 CoFe_2_O_4_) + silver chromite (0.7 Ag_0.5_Cr_2.5_O_4_)] was assayed for its antiviral and cytotoxic activity. Finally, the tested compounds could be attractive and alternative antibacterial compounds that open a new path in chemotherapy.

## Conclusion

The preparation of seven silver nanocomposites using flash auto combustion techniques was synthesized successfully. The morphology of the nanoparticles was investigated using high-resolution transmission electron microscopy (HRTEM), scanning electron microscopy (SEM), and atomic force microscopy to ensure that they were formed in nanosized. The tested compounds (especially 15 N due to the presence of cobalt ferrite and silver chromite) showed promising antibacterial and antifungal activity; at the same time the cytotoxicity was low, and they could be used as efficient therapeutic agents against multidrug-resistant microbes that cause human diseases.

## Data Availability

The data that support the findings of this study are available on request from the corresponding author.
